# Renal protective effects of Porphyra dentate aqueous extract in diabetic mice

**DOI:** 10.7603/s40681-014-0018-x

**Published:** 2014-08-13

**Authors:** Pei-Chun Chao, Cheng-Chin Hsu, Wen-Hu Liu

**Affiliations:** 1 School of Nutrition, Chung Shan Medical University, Taichung, Taiwan; 2 Department of Nutrition, Chung Shan Medical University Hospital, Taichung, Taiwan

**Keywords:** *Porphyra dentate*, Diabetes, Oxidative stress, Hyperlipidemia

## Abstract

Background: Purple laver ((*Porphyra dentate*) is a popular edible seaweed in Asia. This study examined protective effects of extract from purple laver extract (PLE) in diabetic mice.

Methods: Content of carotenoids and anthocyanins in PLE was analyzed. PLE at 0.5 and 1% was supplied for 7 weeks.

Results: PLE was rich in anthocyanins. PLE intake at 0.5 and 1% lowered plasma glucose level (P<0.05); only at 1% raised plasma insulin level, and decreased plasma triglyceride and total cholesterol levels (P<0.05). PLE treatments at 1% lowered hepatic triglyceride and total cholesterol (P<0.05); it reduced renal reactive oxygen species level (P<0.05); retained renal glutathione level, maintaining renal glutathione peroxidase and catalase activities (P<0.05).

Conclusion: *Porphyra dentate* aqueous extract could attenuate diabetic progression via anti-oxidative and lipid lowering effects. This seaweed could be considered as potent healthy food, and used for personalized medicine.

## 1. Introduction

Diabetes is a common chronic disease in Taiwan and other countries. Complications including nephropathy and atherosclerosis contribute to severity of this disease, as well as increase the mortality of patients. Clinical characteristics of these complications include hyperglycemia, hyperlipidemia and oxidation stress [1-3]. Thus, it is vital to control these risk factors in order to delay diabetic deterioration. Diabetics areencouraged to consume more fresh vegetable and fruit in order to obtain phytochemicals like flavonoids, carotenoids and anthocyanins; most of these modify glucose homeostasis and afford anti-oxidative protection [4-6].

*Porphyra dentate* is a seaweed with purple color, also called purple laver or nori, usually consumed as a vegetable in Taiwan, China and Japan. Noda [7] reported purple laver contains many active substances beneficial to human health.Shaw and Liu [8] revealed this plant food as rich in iron. Takenaka et al. [9] reported purple laver as a good source of vitamin B12. So far, less information is available regarding its phytochemical composition. Its purple-red color seems to indicate the presence of carotenoids or anthocyanins. It remains unknown whether this seaweed can provide preventive effect against diabetes. Animal study examined effect of purple laver extract on glycemic control in a mouse model of diabetes. Anti-diabetic benefits were rated by variation of reactive oxygen species (ROS), glutathione (GSH), triglyceride, cholesterol and kactivity of certain enzymes responsible for antioxidant defense in diabetic mice. Our study examined protective effects and actions from *Porphyra dentate* against diabetic progression. Results enhance understanding regarding application of thisplant food.

## 2. Materials and methods

### 2.1. Materials

Fresh purple laver, harvested in Fall 2012, was obtained from farms in Penghu Island, Taiwan. A 50 g edible portion of purple laver was chopped and mixed with 150 mL sterile distilled water at 25°C for 12 hr, then homogenized in a Waring blender. After filtration through a Whatman No. 1 paper, filtrate was freezedried to fine powder.

### 2.2. Determination of carotenoid and anthocyanin

The method of Craft [10] ascertained total carotenoid content at 454 nm by a UV-Vis spectrophotometer, expressed as lutein equivalents. A pH differential method determined total anthocyanin content, expressed as cyanidin-3-glucoside equivalents [11], absorbances at 510 and 700 nm in buffers at pH 1.0 and 4.5 measured.

**Table 1:** Water intake (WI, mL/mouse/day), feed intake (FI, g/mouse/day) and body weight (BW, g/mouse) of normal, diabetic mice consumed normal diet (DM), or 0.5, 1% purple laver extract (PLE) at Week 7. Data are expressed as mean ± SD, n=9.

	Normal	DM	DM+PLE, 0.5%	DM+PLE, 1%
WI	1.9±0.4^a^	5.9±1.0^d^	4.7±0.6^c^	3.5±0.7^b^
FI	2.1±0.5^a^	6.1±0.9^c^	4.4±1.2^b^	3.8±0.8^b^
BW	28.0±1.8^c^	14.2±0.8^a^	16.3±0.7^b^	19.3±1.0^c^

^a-d^Means in a row without a common letter differ, *P*<0.05.

### 2.3. Animals and diets

Male Balb/cA mice, five weeks old, were obtained from National Laboratory Animal Center (National Science Council, Taipei City, Taiwan). Use of mice was reviewed and approved by Chung Shan Medical University animal care committee. To induce diabetes, mice with body weight of 24.0 ± 0.8 g were treated with streptozotocin (50 mg/kg body weight in 0.1 mol/L citrate buffer, pH 4.5) i.p. for 3 consecutive days. Blood glucose level was monitored on Day 10 from the tail vein, using a one-touch blood glucose meter (Lifescan Inc., Milpitas, CA). Mice with fasting blood glucose levels ≥ 14.0 mmol/L were used for this study. After diabetes was induced, mice were divided into several groups of nine.

### 2.4. Experimental design

Powder of purple laver extract (PLE) at 0.5 or 1 g was mixed with 99.5 or 99 g standard powder diet. After seven weeks of supplementation, liver and kidneys from each mouse were collected and weighed. Blood was collected and plasma forthwith separated from erythrocyte. Liver or kidney at 0.1 g was homogenized on ice in 2 mL phosphate buffer saline (PBS, pH 7.2). Protein concentration of plasma or organ homogenate was assayed by commercial kit (Pierce Biotechnology Inc., Rockford, IL) with bovine serum albumin as a standard.

### 2.5. Blood glucose and insulin analyses

Plasma glucose level (mmol/L) was measured by a glucose HK kit (Sigma Chemical Co., St. Louis, MO). Plasma insulin level (nmol/L) was measured by using a rat insulin kit (SRI-13K, Linco Research Inc., St. Charles, MO).

### 2.6. Determination of triglyceride (TG) and total cholesterol (TC) in plasma and liver

Plasma TG and TC were gauged by triglyceride/GB and cholesterol/HP kits (Boehringer Mannheim, Mannheim, Germany), respectively. Liver homogenate (1 mL) was mixed with 2.5 mL chloroform/methanol (2:1, v/v), chloroform layer collected and concentrated. After mixing with 10% Triton X-100 in isopropanol, sample was assayed by Wako Triglyceride E-Test and Total Cholesterol E-Test kits (Wako Pure Chemical, Osaka, Japan).

### 2.7. GSH and ROS levels, glutathione peroxidase (GPX) and catalase activities assay

GSH concentration (nmol/mg protein) in kidney was determined by commercial colorimetric GSH assay kits (OxisResearch, Portland, OR). Method described in Gupta et al. [12] served to measure ROS in kidney. GPX or catalase activity (U/mg protein) in kidney was determined by GPX and catalase assay kits (Calbiochem, EMD Biosciences, Inc., San Diego, CA).

### 2.8. Statistical analyses

Effect of each treatment was analyzed from 9 mice (n=9) in each group. Data were subjected to analysis of variance (ANOVA), differences with *P*<0.05 considered significant.

## 3. Results

**Fig 1:**
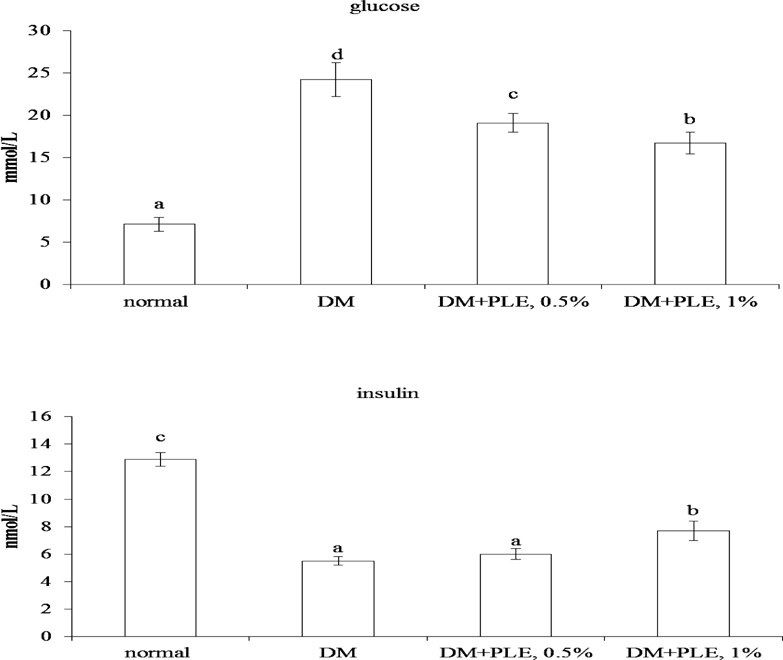
Plasma levels of glucose (mmol/L) and insulin (nmol/L) of normal, diabetic mice consumed normal diet (DM), or 0.5, 1% purple laver extract (PLE) at Week 7. Data are expressed as mean ± SD, n=9. ^a-d^Means among bars without a common letter differ, P<0.05.

Total carotenoid and anthocyanin content in PLE were 144-208 and 1562-1773 mg/g dry weight, respectively. As shown in Table 1, compared with DM control groups, mice with 1% PLE intake had lower water intake, lower feed intake and higher body weight (*P*<0.05). Plasma levels of glucose and insulin are presented in Figure 1. When compared with DM control group, PLE treatments at 0.5 and 1% reduced plasma glucose level (*P*<0.05). Yet PLE treatments only at 1% raised plasma insulin level (*P*<0.05). Table 2 shows PLE treatments at 0.5 and 1% decreasing plasma and hepatic TG levels (*P*<0.05) but not affecting TC level in plasma or liver (*P*>0.05). As shown in Table 3, PLE treatments retained GSH level and decreased ROS level in kidney (*P*<0.05). PLE treatments at 1% maintained GPX and catalase activities in kidney (*P*<0.05).

**Table 2:** TG and TC content in plasma (mmol/L) and liver (mmol/mg protein) in normal, diabetic mice consumed normal diet (DM), or 0.5, 1% purple laver extract (PLE) at Week 7. Data are expressed as mean ± SD, n=9.

	normal	DM	DM+PLE, 0.5%	DM+PLE, 1%
Plasma				
TG	0.39±0.08^a^	0.79±0.12^d^	0.64±0.06^c^	0.42±0.04^b^
TC	0.18±0.06^a^	0.59±0.10^b^	0.51±0.13^b^	0.48±0.08^b^
Hepatic				
TG	1.18±0.14^a^	2.47±0.26^d^	2.02±0.10^c^	1.78±0.09^b^
TC	0.91±0.07^a^	1.49±0.12^b^	1.35±0.16^b^	1.28±0.14^b^

^a-d^Means in a row without a common letter differ, *P*<0.05.

**Table 3:** Level of ROS (nmol/mg protein), GSH (nmol/mg protein) and activity (U/mg protein) of catalase and GPX in kidney from of normal, diabetic mice consumed normal diet (DM), or 0.5, 1% purple laver extract (PLE) at Week 7. Data are expressed as mean ± SD, n=9.

	normal	DM	DM+PLE, 0.5%	DM+PLE, 1%
ROS	0.23±0.09^a^	1.67±0.18^d^	1.26±0.13^c^	0.95±0.07^b^
GSH	11.2±0.25^d^	4.3±0.10^a^	6.0±0.17^b^	7.4±0.2^c^
GPX	17.6±0.19^d^	10.3±0.14^a^	12.0±0.21^b^	13.7±0.15^c^
catalase	15.3±0.08^c^	8.8±0.11^a^	10.1±0.13^b^	10.5±0.22^b^

^a-d^Means in a row without a common letter differ, *P*<0.05.

## 4. Discussion

It is reported that purple laver contains iron and vitamin B_12_ [8, 9]. Our study found purple laver aqueous extract containing carotenoids and anthocyanins. It is reported that the latter possess pharmacological properties and may protect against chronic diseases like diabetes [13,14]. Findings indicate that this seaweed, at least via these phytochemicals, provides nutritional benefits. The presence of anthocyanins also explains the purple-red color of this plant food.

Hyperglycemia is a pivotal factor responsible for development of d iabetic complications [15,16]. In this study, intake of purple laver aqueous extract attenuated hyperglycemia and hypoinsulinemia in diabetic mice. These findings indicated that this plant benefited glycemic control in those diabetic mice, which in turn improved feed intake, water intake and body weight loss. Hyperlipidemia and steatosis are two common clinical characteristics in diabetic subjects [17,18]. Lipid accumulation in blood or organs resulted from diabetes not only affects hepatic functions but also heightens risk of cardiovascular disorder [19,20]. We found that purple laver intake decreased triglyceride accumulation in circulation and liver. It seems that this plant food could promote lipid metabolism under diabetic condition. One possibility was that purple laver intake improved glycemic control and insulin sensitivity, which further enhanced the utility of dietary lipid. Further study must examine its influence upon activity of lipid metabolism-associated enzymes like fatty acid synthases, this to elucidate the actions.

Oxidative stress emanating from hyperglycemia impairs organs functions and facilitates diabetic deterioration [21,22]. Our data agreed with previous studies: oxidative stress present in kidneys of diabetic mice. However, we further found that the intake of purple laver aqueous extract mitigated renal oxidative injury. It is possible that this extract retained renal GSH content, which in turn lowered ROS formation, indicating this extract could spare renal GSH. Likewise, it is reported that intake of purple laver effectively maintains renal activity of GPX and catalase. Purple laver might mediate these enzymes; results imply that it spawns both non-enzymatic and enzymatic anti-oxidative activities under diabetic conditions to diminish oxidative injury. Since this seaweed contains phytochemicals, the observed anti-oxidative protection in diabetic mice could be partially attributed to presence of anthocyanins and/or carotenoids in this aqueous extract.

Our results portend aqueous extract of purple laver attenuating diabetic progression via multiple actions, partially explaining actions of purple laver as medicinal food. Purple laver is popular seaweed in Asia countries; 0.5 or 1% dosage in our study was equal to 90-180 g/day for a 70-kg adult. Based on natural properties and low doses required, applying this seaweed to diabetic cases seems safe and feasible. In sum, our study provided several novel findings to elucidate composition and anti-diabetic e ffect of purple laver, which contains carotenoids and anthocyanins. Aqueous extract of it exhibited hypoglycemic, hypolipidemic and anti-oxidative protection in diabetic mice. These suggest that pepino could be developed as a functional food for anti-diabetic prevention and/or alleviation.

**Declaration of Interest:** Authors declare no conflicts of interest for this work.
